# ITS1 PCR-RFLP Diagnosis and Characterization of *Leishmania* in Clinical Samples and Strains from Cases of Human Cutaneous Leishmaniasis in States of the Mexican Southeast

**DOI:** 10.1155/2014/607287

**Published:** 2014-07-01

**Authors:** Amalia Monroy-Ostria, Abedelmajeed Nasereddin, Victor M. Monteon, Carmen Guzmán-Bracho, Charles L. Jaffe

**Affiliations:** ^1^Escuela Nacional de Ciencias Biológicas, IPN, Carpio y Plan de Ayala, 11340 Mexico City, Mexico; ^2^School of Medicine Department of Microbiology and Molecular Genetics, IMRIC, The Hebrew University of Jerusalem, 9112102 Jerusalem, Israel; ^3^Centro de Investigaciones Biomédicas, Universidad Autonoma de Campeche, Patricio Trueba, 24090 Campeche, Mexico; ^4^Instituto de Diagnóstico y Referencia Epidemiológicos, Francisco P. de Miranda Lomas de Plateros, 01480 Mexico City, Mexico

## Abstract

American cutaneous leishmaniasis includes a spectrum of clinical forms localized cutaneous, diffuse cutaneous, and mucocutaneous leishmaniasis which can be caused by different strains of *Leishmania* belonging to the *L. mexicana* or *L. braziliensis* complexes which may coexist in the same endemic area. We evaluated the PCR-RFLP assay of the ITS1 genes for direct identification of *Leishmania* species in 163 clinical samples and 21 Mexican isolates of *Leishmania*. In relation to the Mexican isolates of *Leishmania* 52% displayed a pattern similar to the *L. (L.) mexicana*, 5% showed a mixed pattern compatible with *L. (L.) mexicana* and *L. (V.) braziliensis*, eight with *L. (L.) amazonensis* and *L. (L.) mexicana*, and one to *L. (V.) braziliensis*. Most of the clinical samples, 109/116 (94%), gave a pattern similar to that of the *L. mexicana*, two clinical samples gave similar patterns to that of *Leishmania* braziliensis, and 5 samples gave patterns that suggest a coinfection of *L. (L.) mexicana* and *L. (V.) braziliensis* or *L. (L.) mexicana* and *L. (L.) amazonensis*. The ITS1 PCR-RFLP assay is a multipurpose tool for diagnosis of *Leishmania* from clinical samples and enables determination of the infecting species of New World *Leishmania* in the field in relatively short time and low cost.

## 1. Introduction

Leishmaniasis is a group of parasitic diseases with worldwide distribution. Cutaneous leishmaniasis (CL) is the most widespread form of leishmaniasis, causing primary localized skin lesions (LCL) that can self-heal, but from which parasites can disseminate to the nasopharyngeal mucosa and cause secondary lesions typical of mucocutaneous leishmaniasis (MCL) or disseminate to the entire body in the form of nodular lesions in diffuse cutaneous leishmaniasis (DCL). World Health Organization estimates a worldwide prevalence of approximately 12 million cases, with an annual mortality rate of 60,000. The size of the population at risk is approximately 350 million [1].

American cutaneous leishmaniasis includes LCL caused by* Leishmania* (*L.*)* mexicana*, DCL caused by* Leishmania* (*L*.)* amazonensis*,* Leishmania* (*L*.)* venezuelensis*, and* Leishmania* (*L.*)* pifanoi*, and MCL caused by members of the* L*.* braziliensis* complex [2].

In endemic regions, multiple species of* Leishmania *may coexist. Identification of the infecting species based on clinical symptoms is difficult, since several species can cause both LCL and MCL. In some villages in Mexico, patients with lesions produced by both* L. braziliensis *and* L. mexicana *complex members can be found as well as patients with LCL and patients with DCL in the same village [3]. Moreover, reports indicate that the response to therapeutic drugs can vary among different species present in the same area [4]. Diagnostic confirmation and correct identification of the* Leishmania *species are important for appropriate species-specific therapeutic as well as epidemiologic studies.

The polymerase chain reaction (PCR) approach was developed as an alternative to existing diagnostic procedures such as direct detection of parasites by microscopic examination of clinical specimens or by cultivation.

Several molecular targets for a diagnostic PCR have been evaluated in* Leishmania *including minicircle kinetoplast DNA (kDNA) [3], the miniexon (spliced leader RNA) gene [5], the gp63 PCR-RFLP [6], and the internal transcribed spacer (ITS) [7–9], among others.

In the present study, as described by Cupolillo et al. [10] and Schönian et al. [11], samples spotted on filter paper and* Leishmania* isolates from patients with cutaneous ulcers suspected of having LC were analyzed by PCR amplification of the internal transcribed spacer 1 genes (ITS1) and restriction fragment length polymorphism (ITS1 PCR-RFLP) for the direct diagnosis of leishmaniasis and parasite identification.

The aim of this study was to look for a diagnostic method for leishmaniasis that combines high sensitivity with species differentiation in the field, in short time and low cost.

## 2. Materials and Methods

### 2.1. Ethical Considerations

Informed consent was obtained from all the adults who participated in the study. Consent for inclusion of young children was obtained from parents or guardians. The protocol of the present study was reviewed and approved by the Ethics Committee of Health Authorities of Calakmul Campeche, Mexico, in agreement with International Ethics Guidelines for Biomedical Research involving human subjects (Norma Oficial Mexicana de Salud: NOM-003-SSA 2-1993), for bleeding human beings for diagnosis and therapeutics.

### 2.2. *Leishmania* Cultures and Clinical Samples

This study was conducted with 21 cultures of* Leishmania* isolated from patients with cutaneous ulcer from different states of Mexico, kindly donated by Instituto de Diagnostico y Referencia, Secretaria de Salud México.

The clinical samples (163) were kindly donated by Centro de Investigaciones Biomédicas, Universidad de Campeche, and Los Servicios de Salud del Municipio de Calakmul Campeche, Mexico. The clinical samples were taken on filter papers or smears from the cutaneous lesions of patients suspected of having CL from different endemic areas of Mexico.

### 2.3. *Leishmania* Reference Strains

*Leishmania (V.) panamensis* (MHOM/CR/87/NEL3),* Leishmania (V.) panamensis* MHOM/PA/72/LS94,* Leishmania (V.) guyanensis *(MHOM/BR/75/M4147),* L. (L.) mexicana *(MHOM/MX/85/SOLIS),* L. (V.) braziliensis* (MHOM/BR/75/M2903), and* L. (L.) amazonensis *(MHOM/BR/73/M2269) reference strains were used as controls. The strains of* Leishmania *were cultured in RPMI medium supplemented with 10% fetal calf serum at 26°C.

### 2.4. DNA Extraction

Each clinical specimen was cut from the filter paper or eluted from the smear and incubated in 250 *μ*L cell lysis buffer for 1 h at 56°C. DNA from* Leishmania* cultures was prepared by centrifuging 10^8^ parasites in the exponential phase of growth at 2000 g for 10 min at 4°C. The DNA was extracted from the pellet using the High Pure PCR template preparation kit (Roche Diagnostics GmbH, Mannheim, Germany), following the manufacturer's instructions. The DNA was stored at −20°C until being used.

### 2.5. PCR Analysis of the Internal Transcribed Spacer 1 (ITS1)

The samples were analyzed for ITS1 PCR using 400 nM primers: LITSR: 5′-CTTG GATCATTTTCCGATG-3′ and L5.8S 5′-TGA TAC CAC TTA TCG CAT T-3′ [12]. The reaction was carried out with the PCR-Ready Supreme mix (Syntezza Bioscience, Jerusalem, Israel) in 25 *μ*L of total reaction. Amplification conditions were as described previously [12]. PCR products (8–15 *μ*L) were digested with* Hae III* enzyme, according to the manufacturer's instructions. The amplicons of about 300–350 bp were analyzed on 1.5% agarose gels and the restriction fragments on 4% agarose gels by electrophoresis at 100 V in 1X Tris-acetate-EDTA buffer (0.04 M Tris acetate and 1 mM EDTA, pH 8) and visualized by UV light after being stained with ethidium bromide (0.3 *μ*g/mL). The GeneRuler DNA ladder Mix (Fermentas, MBI) was used as the DNA molecular marker.

## 3. Results

**Figure 1:**
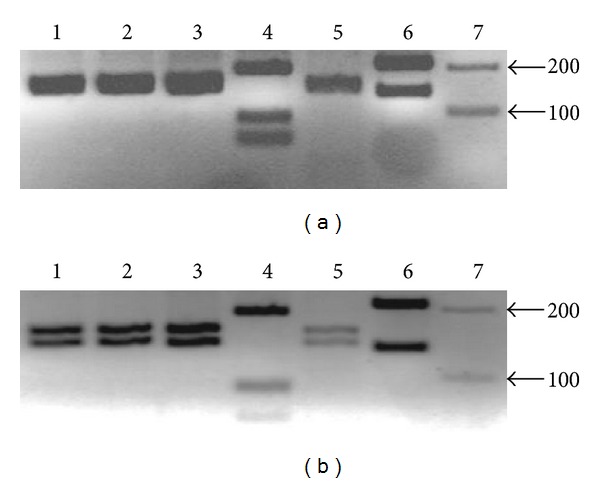
(a) Electrophoresis run at 100 V for 30 min. (b) Electrophoresis run at 199 V for 60 min. PCR-RFLP of the ITS1 of* Leishmania* reference strains. Lane 1:* Leishmania (V.) panamensis *MHOM/CR/87/NEL3; lane 2:* Leishmania (V.) panamensis* MHOM/PA/72/LS9*;* lane 3:* Leishmania (V.) guyanensis *(MHOM/BR/75/M4147); lane 4:* Leishmania (L.) mexicana *(MHOM/MX/85/SOLIS); lane 5:* Leishmania (V.) braziliensis* (MHOM/BR/75/M2903); lane 6:* Leishmania (L.) amazonensis *(MHOM/BR/73/M2269); lane 7: MWMX174* Hae III.*

PCR with specific primers for ITS1 resulted in the amplification of the* Leishmania *reference strains, the Mexican cultures, and the clinical samples giving 300 to 350 bp amplification bands. Restriction of the ITS1 gene amplicons of* L. (V.) panamensis*,* L. (V.) guyanensis*, and* L. (L.) braziliensis* reference strains with the endonuclease* Hae III* generated patterns with two bands of 170 and 150 bp;* L. (L.) amazonensis* generated two bands of 220 and 140 bp; and* L. mexicana *generated three bands of 200, 80, and 40 bp (Figure 1).

**Figure 2:**
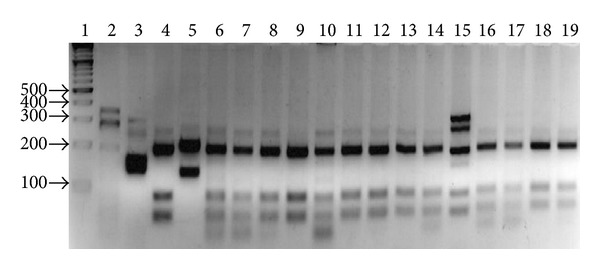
ITS1 PCR-RFPLC of Mexican* Leishmania* cultures. Lane 1: MWMX174* Hae III*; lanes 2 to 19: Mexican cultures of* Leishmania.*

Most of the Mexican isolates of* Leishmania* 11/21 (52%) displayed a restriction pattern of three bands (200, 80, and 40 bp) similar to that of* L. (L.) mexicana* reference strain; nine of these were obtained from patients from Campeche. 1/21 (5%) showed a mixed pattern compatible with* L. (L.) mexicana* and* L. (V.) braziliensis* (lane 15) and one culture with* L. (V.) braziliensis* (lane 3); eight showed a mixed pattern compatible with* L. (L.) amazonensis and L. (L.) mexicana*. In few samples an incomplete digestion can be appreciated (Figure 2) (Table 1); these results were in agreement with a previous study in PCR TS1-RFLP analysis to identify* Leishmania *species in clinical samplesby Rotureau et al. [13] and in the study of PCR diagnosis and characterization of* Leishmania* in clinical samples by Schönian et al. [11].

**Figure 3:**
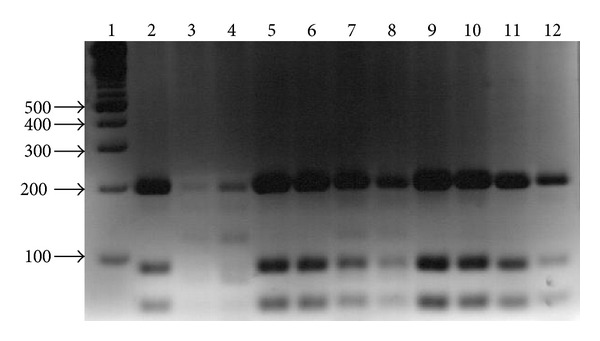
ITS1 PCR-RFLP of Clinical samples. Lane 1: MWMX174* Hae III*; lanes 2 to 12: clinical samples taken on filter paper or smear.

In relation to the clinical samples 116/163 (71%) were amplified, 109/116 (94%) giving a ITS1 PCR-RFLP pattern similar to the* L. (L.) mexicana *reference strain; in seven samples (6%) extra bands of 50 and 25 bp were observed suggesting a coinfection as it was found in the previous study of Hernández-Montes et al. [3] with kDNA PCR analysis of Mexican* Leishmania* species, where they identified in clinical samples both DNA from* L. (L.) mexicana* and* L. (V.) braziliensis*. In lanes 3-4 and 7-8 the pattern of bands of 200, 170, and 140 bp observed suggests the presence of* L. (L.) mexicana*,* L. (V.) braziliensis,* and* L. (L.) amazonensis,* respectively (Figure 3).

## 4. Discussion 

Molecular techniques have proved to be sensitive and powerful tools for detecting* Leishmania *directly in clinical samples as well as for parasite characterization, using the PCR.

Several scientific papers based on ITS analysis have been published on the diagnosis of leishmaniasis and the identification of the* Leishmania *species. Cupolillo et al. [10] evaluated the ITS using restriction patterns of* Leishmania *and* Viannia *rDNA isolates from different hosts and geographical areas, found high levels of intra- and interspecific variation, and showed that the ITS of these genera is evolving fast enough to enable the species to be discriminated.

Interestingly, Schönian et al. [11] established a diagnostic ITS1 PCR-RFLP method using the restriction enzyme* Hae III *for leishmaniasis; it combines high sensitivity for detecting* Leishmania *directly in clinical materials and the ability to identify all medically relevant species groups. On the other hand, Spanakos et al. [14] developed an ITS1 PCR-RFLP method with the endonuclease* Apo I* for the detection and species differentiation of* Leishmania* directly from clinical samples, specific enough to identify all* Leishmania *species commonly encountered in Greece. Slami et al. [15] studied a CL endemic area of Central Iran, by using ITS1 PCR-RFLP analysis for diagnosis of* Leishmania* species in clinical samples and found changes in the profile of* Leishmania *species that could have implications on treatment and/or control strategies. On the other hand El-Beshbishy et al. [16] studies with both ITS1 PCR RFLP and kDNA PCR assays in clinical samples from CL patients from western Saudi Arabia found * L. major* and* L. tropica* and that kDNA PCR had a sensitivity of 90.7% and ITS1 PCR of 70.1%. That facilitated the diagnosis and the species identification using both techniques, whereas parasite culture alone detected 39.2% and smear alone 55.3% of the positive samples. Furthermore Kumar et al. [17], in a CL endemic area of India using ITS1 PCR-RFLP, kDNA PCR, and specific antibody detection, found similar results and* L. tropica* as the causative parasite.

On the other hand Abbasi et al. [18] performed a prospective cohort study on the transmission dynamics of VL in blood samples collected from villagers in the Tahtay Adiabo district of northern Ethiopia combining quantitative real-time kinetoplast DNA/PCR (qRT-kDNA PCR) for detecting small quantity of* Leishmania* parasites (1–10/mL of blood) and sequencing the ITS1 PCR amplicon in order to identify the* Leishmania *species.

On the other hand Rotureau et al. [13], in diagnosis of CL and MCL New World* Leishmania* species using ITS1 PCR RFLP, found that only one digestion with* Rsa I* is required to identify parasites in clinical samples to the species level digestion, but restriction with* Hae III *was not sufficient to distinguish all species in the* Viannia *subgenus, especially* L*. (*V*.)* braziliensis*/*L*. (*V*.)* naiffi *and* L*. (*V*.)* lainsoni*/*L*. (*V*.)* guyanensis.*

In Mexico, Pérez-Vega et al. [19] in Durango State and Ochoa-Diaz et al. [20] in Sinaloa State identified* Leishmania mexicana* in clinical samples with ITS1 PCR RFLP assay.

**Figure 4:**
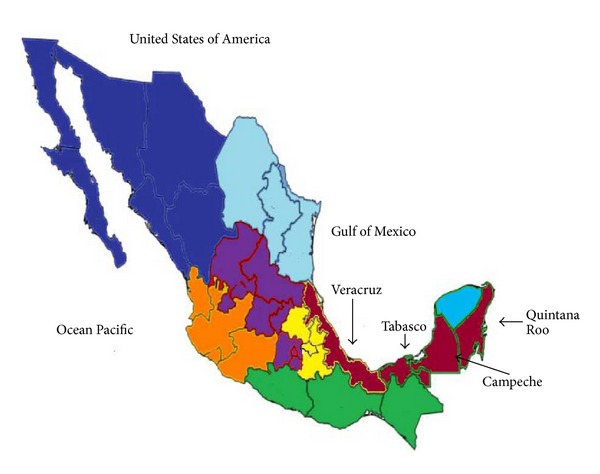
Map of Mexico, showing the endemic regions studied in this work, Veracruz, Tabasco, Campeche, and Quintana Roo.

In the present study following the methodology described by Schönian et al. [11], most of the DCL cases were found in Tabasco and Veracruz States and were caused by* L. (L.) mexicana*, whereas most of the LCL cases produced by* L. (L.) mexicana* belonged to Campeche State as well as the LCL cases caused by* L. braziliensis* complex members (Table 1). All these states are located very close to each other in southeastern Mexico (Figure 4). They all have rain forest areas where CL is endemic. In Tabasco, DCL and LCL coexist; in Campeche it is possible to find LCL caused by* L. (L.) mexicana* or* L. braziliensis* complex members and we were able to detect mixed infections in clinical samples and cultures [3, 21]. This method was very useful for the analysis of the Mexican strains of* Leishmania *and clinical samples because we could perform relatively easy diagnosis and characterization of* Leishmania* species and its possible relationship with the clinical manifestation.

However, this study contradicts the observation of Berzunza-Cruz et al. [22], with ITS restriction patterns and the small subunit rRNA genes of Mexican isolates of* L. mexicana*, finding that all strains showed invariant patterns for both genes.

The PCR-based assays are advantageous over immunological techniques such as enzyme linked immunosorbent assay (ELISA) and immunofluorescence antibody test (IFAT) as host species-specific reagents are not required, which is important in patients with MCL and the immunocompromised ones, in which both give negative serological tests [3]. In particular, in chronic CL patients, who constitute the greater diagnostic challenge due to their low parasite density, PCR assays for the detection of* Leishmania *DNA presented 100% sensitivity. Moreover, the fact that antibodies remain detectable for years after successful treatment makes the application of PCR a necessity [4]. Furthermore, persistent infection has been found in apparently healed scars from MCL patients [7]; the presence of* Leishmania braziliensis *was reported in patients previously treated by immunotherapy or patients being at different stages of treatment and in subjects who had never presented clinical manifestations, but they had lived in endemic areas and migrated to no endemic regions [8].

These results raise questions on (i) the identity of the Mexican strains that displayed restriction patterns that were not compatible with any of the restriction patterns of the reference strains used in this study but suggesting coinfections, (ii) the pathogenicity of these strains, and (iii) their geographic distribution [23]. In order to answer these questions and to establish the identity of the Mexican* Leishmania* strains and their geographical distribution, it would be necessary, following the methodology developed by Van der et al. [24], to analyze several single-locus markers sequencing of the Mexican* Leishmania *strains from most of the endemic areas of Mexico and from patients with all the clinical manifestations of CL (LCL, MCL, and DCL). Furthermore the Reverse Line Blot Hybridization Assay for Molecular Diagnosis of Old World cutaneous Leishmaniasis developed by Nasereddin et al. [25] will be useful, with probes designed from ITS1 PCR amplicons of Mexican strains, for epidemiological studies where a large number of samples need to be screened, in order to test potential reservoir hosts and vectors and for epidemiological surveillance.

## 5. Conclusion

The ITS1PCR-RFLP assay analyzed in this work was a valuable multipurpose tool for diagnosis directly from clinical samples without parasite isolation and enables determination of the infecting species of New World* Leishmania* in the field in a relatively short time. The ITS1 PCR-RFLP assay is recommended for the reliable characterization of* Leishmania* species mainly in endemic areas where the presence of multiple species of* Leishmania* overlapping clinical pictures demands simultaneous species identification at a relative low cost. Although in areas where both* Leishmania* and* Viannia* subgenus species are present, ITS1 PCR-RFLP must be combined with kDNA PCR in order to improve the sensitivity for diagnosis of CL or MCL.

## Figures and Tables

**Table 1:** ITS1 PCR-RFLP of isolates of *Leishmania* analyzed in this study.

Number	Code	Origin	Pathology	ITS1 PCR-RFLP (bp)	*Leishmania* species
1	MHOM/MX/84/ISET GS	Tabasco	DCL	(220, 200, 140, 40)	*L. am + L. mex *
2	MHOM/MX/88/HRC MC	Tabasco	LCL	(200, 80.40)	*L. mexicana *
3	MHOM/MX/88/HRC JS	Tabasco	LCL	(200, 80.40)	*L. mexicana *
4	AMG	Tabasco	DCL	(220, 200, 140, 40)	*L. am + L. mex *
5	HC	Tabasco	LCL	(220, 200, 140, 40)	*L. am + L. mex *
6	L.527	Tabasco	LCL	(220, 200, 140, 40)	*L. am + L. mex *
7	MHOM/MX/85/ISET HF	Veracruz	DCL	(220, 200, 140, 40)	*L. am + L. mex *
8	MHOM/MX/92/INDRE AM	Veracruz	DCL	(220, 200, 140, 40)	*L. am + L. mex *
9	LVER	Veracruz	DCL	(220, 200, 140, 40)	*L. am + L. mex *
10	MHOM/MX/83/UAVY	Q. Roo	LCL	(200, 80.40)	*L. mexicana *
11	MHM/MX/06/ENCB/MIC	Campeche	LCL	(200, 80.40)	*L. mexicana *
12	MHM/MX/06/ENCB CDL	Campeche	LCL	(200, 80.40)	*L. mexicana *
13	MHM/MX/06/ENCB FDL	Campeche	LCL	(200, 80.40)	*L. mexicana *
14	MHM/MX/07/ENCB NDM	Campeche	LCL	(200, 80.40)	*L. mexicana *
15	RMA	Campeche	LCL	(200, 170, 150, 80, 40)	*L. mex + L. bra *
16	REP	Campeche	LCL	(220, 200, 140, 40)	*L. am + L. mex *
17	FAD	Campeche	LCL	(200, 80.40)	*L. mexicana *
18	A MJ	Campeche	LCL	(200, 80.40)	*L. mexicana *
20	DON	Campeche	LCL	(200, 80.40)	*L. mexicana *
20	L.528	Campeche	LCL	(200, 80.40)	*L. mexicana *
21	CR	Campeche	LCL	(170, 150)	*L. braziliensis *

*L. am + L. mex: L. (L.) amazonensis + L. (L.) mexicana.*

*L. mexicana: L. (L.) mexicana.*

*L. mex + L. bra: L. (L.) mexicana + L. (V.) braziliensis.*
